# Blood pressure fragmentation as a new measure of blood pressure variability: association with predictors of cardiac surgery outcomes

**DOI:** 10.3389/fphys.2024.1277592

**Published:** 2024-02-09

**Authors:** Madalena D. Costa, Valluvan Rangasamy, Alkananda Behera, Priyam Mathur, Tanvi Khera, Ary L. Goldberger, Balachundhar Subramaniam

**Affiliations:** ^1^ Margret and H. A. Rey Institute for Nonlinear Dynamics in Medicine, Department of Medicine, Beth Israel Deaconess Medical Center, Harvard Medical School, Boston, MA, United States; ^2^ Sadhguru Center for a Conscious Planet, Department of Anesthesia, Critical Care and Pain Medicine, Beth Israel Deaconess Medical Center, Harvard Medical School, Boston, MA, United States

**Keywords:** aging, alternans, beat-to-beat blood pressure variability, blood pressure fragmentation, cardiac surgical risk prediction, ICU length of stay

## Abstract

**Background:** Fluctuations in beat-to-beat blood pressure variability (BPV) encode untapped information of clinical utility. A need exists for developing new methods to quantify the dynamical properties of these fluctuations beyond their mean and variance.

**Objectives:** Introduction of a new beat-to-beat BPV measure, termed blood pressure fragmentation (BPF), and testing of whether increased preoperative BPF is associated with (i) older age; (ii) higher cardiac surgical risk, assessed using the Society of Thoracic Surgeons’ (STS) Risk of Morbidity and Mortality index and the European System for Cardiac Operative Risk Evaluation Score (EuroSCORE II); and (iii) longer ICU length of stay (LOS) following cardiac surgery. The secondary objective was to use standard BPV measures, specifically, mean, SD, coefficient of variation (CV), average real variability (ARV), as well a short-term scaling index, the detrended fluctuation analysis (DFA) ⍺_1_ exponent, in the same type of analyses to compare the results with those obtained using BPF.

**Methods:** Consecutive sample of 497 adult patients (72% male; age, median [inter-quartile range]: 67 [59–75] years) undergoing cardiac surgery with cardiopulmonary bypass. Fragmentation, standard BPV and DFA ⍺_1_ measures were derived from preoperative systolic blood pressure (SBP) time series obtained from radial artery recordings.

**Results:** Increased preoperative systolic BPF was associated with older age, higher STS Risk of Morbidity and Mortality and EuroSCORE II values, and longer ICU LOS in all models. Specifically, a one-SD increase in systolic BPF (9%) was associated with a 26% (13%–40%) higher likelihood of longer ICU LOS (>2 days). Among the other measures, only ARV and DFA ⍺_1_ tended to be associated with longer ICU LOS. However, the associations did not reach significance in the most adjusted models.

**Conclusion:** Preoperative BPF was significantly associated with preoperative predictors of cardiac surgical outcomes as well as with ICU LOS. Our findings encourage future studies of preoperative BPF for assessment of health status and risk stratification of surgical and non-surgical patients.

## Introduction

Much effort has been directed at extracting clinical information from measures of arterial blood pressure variability (BPV). Most research has focused on the predictive utility of variations in intermittent blood pressure (BP) measurements obtained minutes to hours apart, or at even longer time intervals (e.g., visit-to-visit) (Parati et al., 2015; Parati et al., 2018; Packiasabapathy et al., 2020; Cremer et al., 2021; Dasa et al., 2021; Harding et al., 2021; Daniel et al., 2022; Heshmatollah et al., 2022; Liu et al., 2022; Wong et al., 2022). In general, the results lend support to the notion that increased BPV is a risk factor for cardiovascular disease (Stevens, et al., 2016; Tully et al., 2020; Liu et al., 2022), cognitive dysfunction (Zhou et al., 2019; Daniel et al., 2022; Guo et al., 2023; Mahinrad et al., 2023) and all-cause mortality (Dasa et al., 2021). One of the hypothesized mediators of these associations is vascular stiffness (Schillaci et al., 2012; Cremer et al., 2021; Heshmatollah et al., 2022).

The utility of BPV assessment based on intermittent measurements raises the possibility of extracting complementary information from continuous BP monitoring (Parati et al., 2023). A classical inspiration for analysis of beat-to-beat BP time series is the recognition that BP (pulsus) alternans (i.e., alternation in the amplitude of systolic BP during sinus rhythm) is associated with chronic (usually severe) heart failure (Traube, 1872; Surawicz and Fisch, 1992). A number of recent studies also indicate that information encoded in continuous BPV may, in fact, be clinically useful for cardiovascular risk assessment (Henriques et al., 2018; Webb et al., 2018; Rangasamy et al., 2020; Bakkar et al., 2021).

The purpose of this report is to introduce a new measure of continuous BPV, termed blood pressure fragmentation (BPF), developed as a generalization of pulsus alternans, and to provide evidence of its potential translational value in the prediction of cardiac surgical outcomes. To the extent that pulsus alternans and BPF have similar etiologies and both are manifestations of cardiovascular instability, we hypothesized that BPF would be higher in older individuals and in those with poorer health status, the latter quantified by preoperative risk scores. Since, preoperative risk is in turn associated with ICU length of stay (LOS), a surrogate measure of long-term surgical outcome (Moitra et al., 2016; Trivedi et al., 2019), we further hypothesized that increased preoperative BPF would be associated with longer ICU LOS. Beyond assessing these relationships, we sought to determine whether BPF added value to preoperative risk scores in the prediction of LOS ICU. Answering this question is pertinent for providing evidence of the translational utility of BPF.

Finally, we sought to compare the performances of the newly proposed index with those of traditional (mean, SD, coefficient of variation [CV], and average real variability [ARV] (Mena et al., 2005)) and nonlinear (detrended fluctuation analysis [DFA] ⍺_1_ (Peng et al., 1994)) metrics.

## Methods

### Study population

The study population has been previously described (Henriques et al., 2018; Rangasamy et al., 2020). Data for this cohort study derive from an observational investigation funded by the National Institutes of Health (R01GM098406) conducted from January 2013 to September 2016. The study was performed in accord with the Strengthening the Reporting of OBservational Studies in Epidemiology (STROBE) statement (von Elm et al., 2007). Briefly, after obtaining Institutional Review Board (Beth Israel Deaconess Medical Center, Boston, MA) approval and informed verbal consent, 497 adult patients undergoing cardiac surgery with cardiopulmonary bypass were sequentially enrolled. Data related to patient characteristics, surgery, anesthesia, and hemodynamics were collected from institutional databases and the Anesthesia Information Management System (AIMS; Philips Medical, Andover, MA). Surgical risk was obtained from two standard instruments: the Society of Thoracic Surgeons’ (STS) Risk of Morbidity and Mortality index, hereafter abbreviated as STS, and the European System for Cardiac Operative Risk Evaluation Score (EuroSCORE II). Creatinine clearance was estimated using the Modification of Diet in Renal Disease (MDRD) equation. To restrict the analyses to those in sinus rhythm, we excluded participants with paroxysmal or persistent atrial flutter or atrial fibrillation during final preoperative preparation (*n* = 44) as well as those with an electronic pacemaker (n = 10). Note that pulsus alternans (of which BPF is a generalization) has only been defined for sinus beats. Furthermore, focusing on the more uniform group of those in sinus rhythm excludes the possibility of our results being influenced by differences in BPF solely attributable to AF/pacing. Additionally, we excluded participants (*n* = 9) who died within 30 days of surgery, since 33% of them (3 out of 9) had a “misleadingly” short (≤2 days) ICU LOS. Analyses including these subjects are presented in the Supplementary Material.

### Radial arterial blood pressure waveform recording

After sedation with midazolam and before anesthetic induction, a 20-gauge catheter was inserted into a radial artery for recording continuous arterial BP waveforms (Philips Medical, Andover, MA). The recordings were obtained with a sampling frequency of 125 Hz and a 12-bit amplitude resolution. Patients (*n* = 58) whose preoperative recordings were shorter than 10 min and/or of insufficient quality for extraction of the systolic blood pressure (SBP) time series were excluded. The final sample size of our analytical cohort was 378 (436–58). The participants excluded were approximately 1 year older and had slightly higher surgical risk (median [inter-quartile range] EuroSCORE II: 2.19 [1.21–4.23] *versus* 1.95 [1.15–4.38]; STS: 14.2 [8.67–20.6] *versus* 12.0 [7.9–20.1] than those in the final analytical cohort. The median (inter-quartile range) duration of the recordings was 24 min (18–41 min). By construct, the STS score was only available for patients who underwent CABG and/or valve replacement/repair procedures (*n* = 340).

### Arterial blood pressure waveform analysis

The time series of SBP were extracted from the continuous arterial BP waveforms (Zong et al., 2003). Values associated with premature beats as well as those below 60 mmHg or above 210 mmHg were excluded. The dynamical indices were derived from the SBP time series. In these analyses, we used the entire time series for recordings lasting ≤1 h or the segment that corresponded to the 1-h period preceding anesthetic induction in the case of recordings lasting >1 h.

### Blood pressure fragmentation (BPF) analysis

Fragmentation was computed from the preoperative SBP time series using a metric based on counts of increases, decreases and no-changes in SBP values from one beat to the next.

**FIGURE 1:**
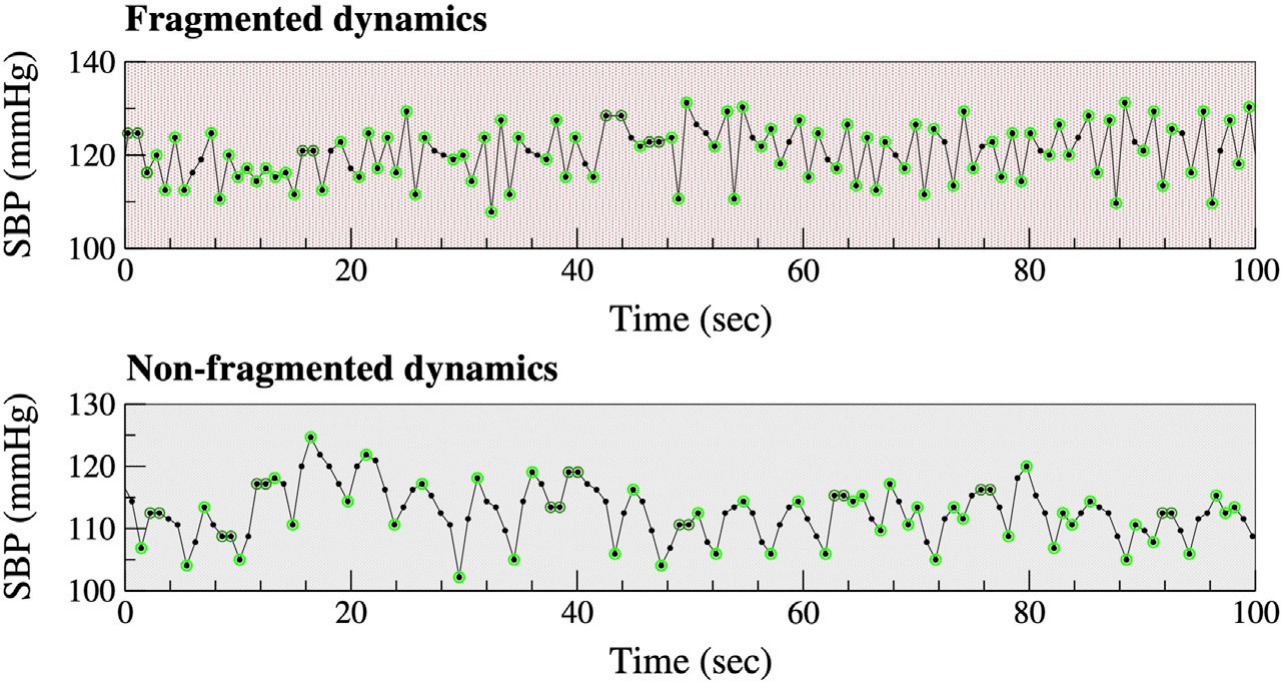
Fragmented *versus* non-fragmented beat-to-beat SBP time series. Light and dark green circumferences around SBP data points highlight “hard” and “soft” inflection points, respectively (see text). The oscillations in the bottom graph (non-fragmented dynamics) have a cycle length of approximately 5 s. These oscillations (∼12/min) are due to respiration. Abbreviation: SBP, systolic blood pressure.

Let SBP_i_ represent the SBP value of a given cardiac pulse and let ∆SBP_i_, defined as SBP_i+1_—SBP_i_, represent the change in SBP from one beat to the next. Increase, decrease and no-change in beat-to-beat SBP are defined, as ∆SBP_i_ >0, ∆SBP_i_ <0, and ∆SBP_i_ = 0, respectively. The resolution of the SBP waveforms was approximately 1 mmHg (more precisely, 0.94 mmHg). Thus, an increase (decrease) in SBP meant that SBP augmented (declined) by more than 1 mmHg from one beat to the next. Decreases in SBP preceded by increases in SBP, as well as increases in SBP preceded by decreases in SBP, are termed “hard inflection points” (Figure 1, light green circles). Decreases (or increases) in SBP preceding or following intervals in which SBP does not change are termed “soft inflection points” (Figure 1, dark green circles). The percentage of all (hard and soft) inflection points, abbreviated PIP, quantifies the degree of BPF. More fragmented time series have higher PIP values (Costa et al., 2017a). Of note, PIP is independent of the amplitude of the fluctuations in SBP time series. In other words, this metric is not affected by how much SBP increases or decreases but only by whether SBP increases or decreases by more than 1 mmHg.

### Standard blood pressure variability (BPV) analysis

The following BPV metrics were calculated: mean, SD, CV and ARV (Mena et al., 2005). The latter is the average of the absolute difference between consecutive SBP values. While SD and CV are measures of overall (both high-frequency and low-frequency) variability, ARV is a measure of “local” (i.e., high-frequency) variability. Note that in contrast to the fragmentation metric, BPV metrics depend on the amplitude of the fluctuations in SBP values.

### Nonlinear (short-term scaling) analysis

Short-term (<12 data points) correlations were quantified by the DFA ⍺_1_ exponent (Peng et al., 1994). The method is based on the assessment of the slope of the regression line of the log-log graph of the fluctuation function, F(n), *versus* the number of BP measurements, n.

### Hypotheses

We hypothesized that both higher BPF and standard BPV metrics would be associated with older age and worse prognosis, namely, higher STS and EuroSCORE II values and longer surgical ICU LOS. Additionally, we anticipated that higher DFA ⍺_1_ values (i.e., values closer to 1.5), consistent with more graduated (smoother) changes in SBP over short time scales (<12 beats) would be associated with younger age, lower STS and EuroSCORE II values and shorter surgical ICU LOS.

### Statistical analysis

Variables were summarized as mean ± SD, unless otherwise specified. Differences in baseline characteristics between those with short and long LOS (defined below) in the surgical ICU were evaluated using the Chi-squared and Mann-Whitney tests for categorical and continuous variables, respectively (Table 1).

**TABLE 1:** Demographic, selected preoperative clinical and blood pressure dynamical characteristics of study groups.

Variable	All	Short ICU LOS	Long ICU LOS	*p* value
	N = 378	N = 198	N = 180	
Age (yrs)	67 [59–75]	65 [57–72]	69 [61–77]	**< 0.001**
Sex: Male	271 (72%)	152 (77%)	119 (66%)	**0.022**
Body Mass Index (kg/m^2^)	28.3 [25.4–32.0]	28.7 [25.6–32.0]	28.2 [25.3–32.5]	0.574
Hematocrit (%)	39.3 [35.7–42.3]	40.1 [ 36.0–42.6]	38.2 [35.2–41.7]	**0.013**
Creatinine Clearance MDRD (ml/min/1.73 m^2^)	72.6 [58.2–85.5]	75.5 [61.6–90.4]	68.3 [52.7–82.5]	**< 0.001**
Albumin (g/dL)	4.1 [3.8–4.5]	4.2 [3.9–4.5]	4.1 [3.8–4.4]	**0.005**
EuroSCORE II (%)	1.95 [1.15–4.38]	1.69 [1.01–2.96]	2.82 [1.41–5.67]	**< 0.001**
STS Morbidity/Mortality (%)*	12.0 [7.9–20.1]	11.0 [7.0–16.4]	14.5 [9.0–25.0]	**< 0.001**
Surgery type				0.118
CABG	196 (52%)	109 (55%)	87 (48%)	
CABG + Valve	65 (17%)	26 (13%)	39 (22%)	
Valve	79 (21%)	45 (23%)	34 (19%)	
Other	38 (10%)	18 (9%)	20 (11%)	
NYHA				0.974
I	43 (11%)	23 (12%)	20 (11%)	
II	168 (44%)	88 (44%)	80 (44%)	
III	112 (30%)	57 (29%)	55 (31%)	
IV	55 (15%)	30 (15%)	25 (14%)	
BPF (%)	62.4 [56.2–67.8]	60.6 [54.4–65.9]	64.2 [57.5–70.6]	**< 0.001**
Mean SBP (mmHg)	136 [122–152]	135 [122–151]	138 [122–153]	0.541
SD (mmHg)	7.24 [5.85–9.26]	7.16 [5.85–9.02]	7.34 [5.85–9.43]	0.909
CV	0.053 [0.043–0.066]	0.053 [0.043–0.066]	0.054 [0.042–0.065]	0.703
ARV (mmHg)	2.97 [2.25–3.86]	2.94 [2.21–3.76]	2.99 [2.26–4.08]	0.264
DFA ⍺_1_	1.11 [0.92–1.27]	1.14 [0.96–1.31]	1.07 [0.86–1.25]	**0.009**

The values for continuous variables are the median and [inter-quartile range]. Values for binary variables are number of participants and (their percentage) in the population/sub-populations. Statistically significant *p* value are highlighted in bold. Abbreviations: ARV, average real variability; BPF, blood pressure fragmentation; CABG, coronary artery bypass graft; CV, coefficient of variation; DFA, detrended fluctuation analysis; EuroSCORE, European System for Cardiac Operative Risk Evaluation; ICU, intensive care unit; LOS, length of stay; MDRD, modification of diet in renal disease equation; NYHA, New York Heart Association; SBP, systolic blood pressure; SD, standard deviation; and STS, Society of Thoracic Surgeons.

*The STS Morbidity/Mortality risk score was calculated for all patients who qualified.

**FIGURE 2:**
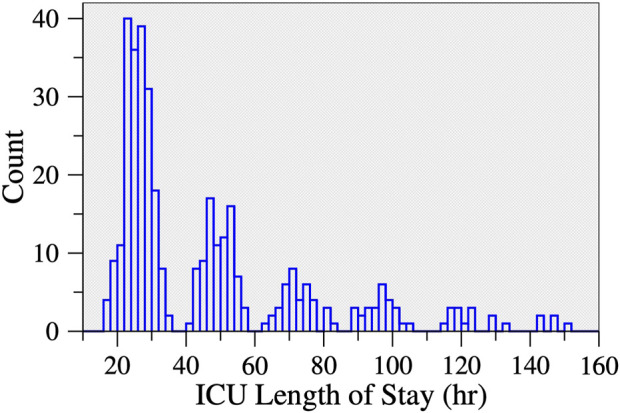
Histogram of ICU length of stay following cardiac surgery. There were 13 participants who stayed >160 h (not shown). Approximately half of the participants (52.4%) stayed < 40 h.

Surgical ICU LOS was analyzed both as a continuous and a binary variable. The latter was motivated by the multimodal nature of the distribution of ICU LOS (Figure 2). Based on this distribution, the cut-off used for the dichotomization of ICU LOS was 40 h. The group of patients who stayed ≤40 h in the surgical ICU (52% of the cohort) was labeled as having a “short” LOS. The other group (48% of the cohort) was labeled as having a “long” LOS. Of note, the multimodal nature of the distribution of ICU LOS was expected. Such distribution is attributable to the daily nature of discharges from the ICU, a process that is not continuous but depends on the patients’ status and to a certain extent on staffing shifts and bed availability for transfer to the wards.

The variables with skewed distributions, STS, EuroSCORE II and ICU LOS were log-transformed. All variables were standardized. The associations of preoperative SBP metrics with cross-sectional age (Table 2) and the two surgical risk indices were evaluated using univariable linear regression models with robust standard errors (Table 3). The associations between each one of the preoperative SBP metrics and ICU LOS were evaluated using linear regression models with robust standard errors. The results for unadjusted, minimally adjusted (by age and sex) and more adjusted (by the STS or EuroSCORE II) models are presented in Table 4. Since STS and EuroSCORE II are comprehensive scores, encompassing a wide array of variables related to patient demographic, medical history, surgical details, laboratory values, physiological parameters and functional status, these models were not adjusted for individual risk factors. The F-test was used to compare the performance (quantified by the R-squared values) of two nested regression models.

**TABLE 2:** Univariable linear regression analysis of the associations between preoperative SBP dynamical metrics and age.

Variable	ß	95% CI		*p* value
BPF (%)	0.34	0.23	0.44	**< 0.001**
Mean SBP (mmHg)	0.11	0.01	0.21	**0.039**
SD (mmHg)	−0.02	−0.12	0.09	0.733
CV	−0.05	−0.16	0.05	0.313
ARV (mmHg)	0.11	0.00	0.21	**< 0.001**
DFA ⍺_1_	−0.23	−0.34	−0.12	**< 0.001**

The values shown are the regression coefficients (ß) and 95% CIs, for standardized variables. Statistically significant *p* values are highlighted in bold. Abbreviations: ARV, average real variability; BPF, blood pressure fragmentation; CI, confidence interval; CV, coefficient of variation; DFA, detrended fluctuation analysis; EuroSCORE, European System for Cardiac Operative Risk Evaluation; LOS, length of stay; SBP, systolic blood pressure; SD, standard deviation; and STS, Society of Thoracic Surgeons.

**TABLE 3:** Univariable linear regression analysis of the associations between preoperative SBP dynamical metrics and the surgical risk scores.

Variable	STS Morbidity/Mortality				EuroSCORE II			
	ß	95% CI		*p* value	ß	95% CI		*p* value
BPF (%)	0.39	0.30	0.49	**< 0.001**	0.34	0.24	0.44	**< 0.001**
Mean SBP (mmHg)	−0.03	−0.15	0.08	0.546	−0.03	−0.14	0.08	0.556
SD (mmHg)	−0.10	−0.21	0.02	0.094	−0.05	−0.16	0.07	0.426
CV	−0.07	−0.18	0.04	0.218	−0.03	−0.14	0.09	0.653
ARV (mmHg)	0.19	0.10	0.28	**< 0.001**	0.18	0.08	0.28	**< 0.001**
DFA ⍺_1_	−0.47	−0.56	−0.38	**< 0.001**	−0.44	−0.52	−0.35	**< 0.001**

The values shown are the regression coefficients (ß) and 95% CIs, for standardized variables. Statistically significant *p* values are highlighted in bold. Abbreviations: ARV, average real variability; BPF, blood pressure fragmentation; CI, confidence interval; CV, coefficient of variation; DFA, detrended fluctuation analysis; EuroSCORE, European System for Cardiac Operative Risk Evaluation; LOS, length of stay; SBP, systolic blood pressure; SD, standard deviation; and STS, Society of Thoracic Surgeons.

**TABLE 4:** Unadjusted and adjusted linear regression analysis of the associations between each preoperative SBP dynamical metric and postoperative number of hours in the ICU.

A. Sub-group with STS (N = 340)															
	Model 0: Unadjusted					Model 1: Age and Sex					Model 2: STS				
Variable	ß	95% CI		*p* value	R-squared	ß	95% CI		*p* value	R-squared	ß	95% CI		*p* value	R-squared
BPF (%)	0.21	0.11	0.30	**< 0.001**	0.045	0.16	0.04	0.27	**0.007**	0.066	0.10	0.00	0.20	**0.046**	0.110
Mean SBP (mmHg)	0.02	−0.10	0.14	0.705	0.001	0.00	−0.12	0.12	0.953	0.043	0.03	−0.08	0.14	0.548	0.102
SD (mmHg)	−0.04	−0.13	0.05	0.423	0.001	−0.03	−0.12	0.06	0.473	0.044	−0.01	−0.10	0.08	0.861	0.101
CV	−0.05	−0.14	0.04	0.250	0.003	−0.04	−0.13	0.05	0.368	0.045	−0.03	−0.12	0.06	0.466	0.102
ARV (mmHg)	0.15	0.03	0.27	**0.016**	0.024	0.12	−0.00	0.24	0.057	0.058	0.10	−0.02	0.21	0.110	0.110
DFA ⍺_1_	−0.17	−0.28	−0.05	**0.005**	0.029	−0.11	−0.24	−0.02	0.086	0.055	−0.03	−0.14	0.09	0.612	0.101
STS (%)	0.30	0.20	0.41	**< 0.001**	0.101										
B. Group with EuroSCORE II (N = 378)															
	Model 0: Unadjusted					Model 1: Age and Sex					Model 2: EuroSCORE II				
Variable	ß	95% CI		*p* value	R-squared	ß	95% CI		*p* value	R-squared	ß	95% CI		*p* value	R-squared
BPF (%)	0.21	0.12	0.31	**< 0.001**	0.046	0.18	0.07	0.29	**0.001**	0.060	0.15	0.05	0.24	**0.004**	0.081
Mean SBP (mmHg)	0.04	−0.08	0.16	0.513	0.002	0.03	−0.09	0.14	0.654	0.033	0.05	−0.06	0.16	0.403	0.065
SD (mmHg)	−0.04	−0.12	0.05	0.423	0.001	−0.04	−0.12	0.05	0.430	0.034	−0.02	−0.11	0.06	0.586	0.063
CV	−0.06	−0.15	0.03	0.188	0.003	−0.05	−0.14	0.03	0.229	0.035	−0.05	−0.14	0.04	0.242	0.065
ARV (mmHg)	0.18	0.03	0.32	**0.016**	0.032	0.16	0.01	0.31	**0.042**	0.057	0.14	−0.01	0.29	0.077	0.081
DFA ⍺_1_	−0.17	−0.29	−0.06	**0.004**	0.030	−0.13	−0.27	0.00	**0.045**	0.049	−0.08	−0.21	0.05	0.224	0.068
EuroSCORE II (%)	0.25	0.15	0.35	**< 0.001**	0.062										

The values shown are the regression coefficients (ß) and 95% CIs, for standardized variables. Separate models were fitted for each of the different BPV, indicators. The adjustments were age and sex in Model 1, and STS, or EuroSCORE II, in Model 2. Statistically significant *p* values are highlighted in bold. Abbreviations: ARV, average real variability; BPF, blood pressure fragmentation; CI, confidence interval; CV, coefficient of variation; DFA, detrended fluctuation analysis; EuroSCORE, European System for Cardiac Operative Risk Evaluation; ICU, intensive care unit; LOS, length of stay; SBP, systolic blood pressure; SD, standard deviation; and STS, Society of Thoracic Surgeons.

The associations of each of the preoperative SBP dynamical metrics with long ICU LOS (binary variable) were quantified using modified Poisson regression models via generalized estimating equations (Zou, 2004; Yelland et al., 2011). These models utilize a log link function to allow for estimation of relative risks (RR). The same adjustments as those described above were used in these analyses. The RR values presented in Table 5 are for a one-SD increase in the value of the independent variables. The analyses with the STS risk index were restricted to the 340 (out of 378) patients with values for this variable. Statistically significant *p*-values (<0.05) are highlighted in bold in the tables.

**TABLE 5:** Unadjusted and adjusted modified Poisson regression analyses of the associations between each preoperative SBP dynamical metric and long ICU LOS (>40 h).

A. Sub-group with STS Risk Score (N = 340)												
	Model 0: Unadjusted				Model 1: Age and Sex				Model 2: STS			
Variable	RR	95% CI		*p* value	RR	95% CI		*p* value	RR	95% CI		*p* value
BPF (%)	1.26	1.13	1.40	**< 0.001**	1.18	1.05	1.32	**0.006**	1.16	1.03	1.30	**0.012**
Mean SBP (mmHg)	1.01	0.91	1.13	0.833	0.99	0.88	1.10	0.805	1.02	0.93	1.13	0.640
SD (mmHg)	0.95	0.84	1.06	0.362	0.95	0.85	1.07	0.410	0.98	0.87	1.10	0.696
CV	0.93	0.83	1.05	0.225	0.95	0.84	1.06	0.341	0.95	0.85	1.06	0.380
ARV (mmHg)	1.11	1.01	1.22	**0.035**	1.06	0.97	1.17	0.196	1.06	0.97	1.16	0.220
DFA ⍺_1_	0.88	0.79	0.98	**0.021**	0.95	0.85	1.07	0.377	1.00	0.88	1.12	0.941
STS (%)	1.30	1.17	1.44	**< 0.001**								
B. Group with EuroSCORE II (N = 378)												
	Model 0: Unadjusted				Model 1: Age and Sex				Model 2: EuroSCORE II			
Variable	RR	95% CI		*p* value	RR	95% CI		*p* value	RR	95% CI		*p* value
BPF (%)	1.24	1.13	1.37	**< 0.001**	1.18	1.06	1.32	**0.003**	1.15	1.03	1.28	**0.010**
Mean SBP (mmHg)	1.03	0.93	1.15	0.569	1.01	0.91	1.12	0.817	1.04	0.94	1.15	0.406
SD (mmHg)	0.96	0.86	1.07	0.470	0.96	0.87	1.07	0.497	0.98	0.88	1.09	0.654
CV	0.94	0.84	1.05	0.252	0.95	0.85	1.05	0.320	0.95	0.85	1.05	0.306
ARV (mmHg)	1.11	1.02	1.21	**0.018**	1.08	0.99	1.18	0.086	1.06	0.97	1.16	0.195
DFA ⍺_1_	0.86	0.78	0.95	**0.004**	0.91	0.82	1.01	0.079	0.97	0.87	1.08	0.565
EuroSCORE II (%)	1.30	1.19	1.42	**< 0.001**								

Values presented are the relative risk and 95% CI, for standardized variables. Separate models were fitted for each of the different BPV, indicators. The adjustments were age and sex in Model 1, and STS, or EuroSCORE II, in Model 2. Statistically significant *p* values are highlighted in bold. Abbreviations: ARV, average real variability; BPF, blood pressure fragmentation; CI, confidence interval; CV, coefficient of variation; DFA, detrended fluctuation analysis; EuroSCORE, European System for Cardiac Operative Risk Evaluation; ICU, intensive care unit; LOS, length of stay; RR, relative risk; SD, standard deviation; SBP, systolic blood pressure; SD, standard deviation; STS, Society of Thoracic Surgeons.

## Results

### Characteristics of participants with long versus short ICU LOS

**FIGURE 3:**
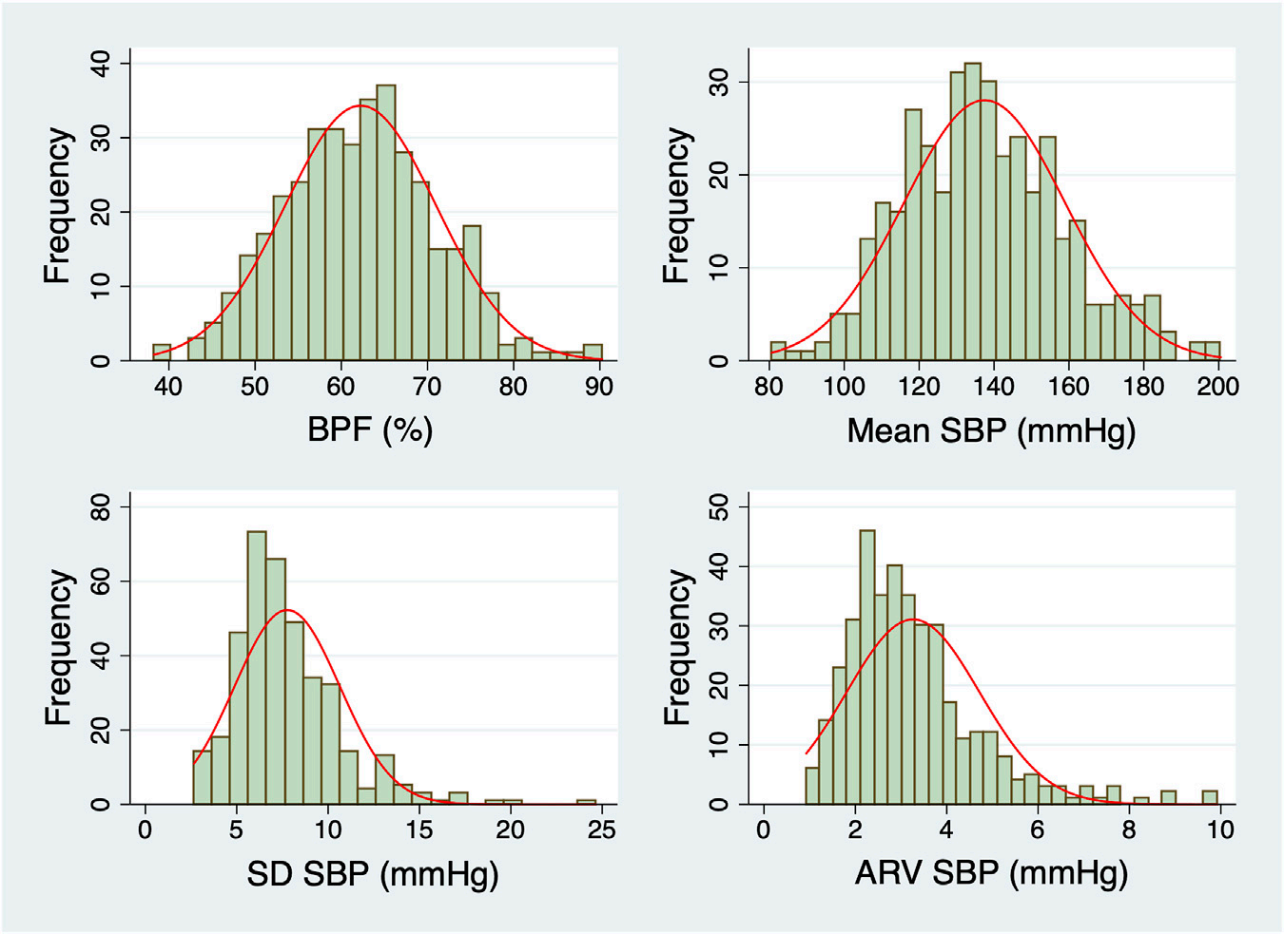
Histograms of the dynamical metrics of preoperative SBP. Normal distributions with the same mean and SD as the data (red lines) are overlaid on each histogram. Abbreviations: ARV, average real variability; BPF, blood pressure fragmentation; SBP, systolic blood pressure; SD, standard deviation.

Participants with long ICU LOS tended to be older (median age: 69 *versus* 65 years), had lower preoperative hematocrit, albumin and estimated creatinine clearance values and higher surgical risk scores (Table 1). The percentage of participants who underwent combined CABG and valve surgeries was higher in the group with long LOS than in the group with short LOS (22% *versus* 13%). Preoperative BPF was significantly higher in those with long ICU LOS (Table 1). The DFA ⍺_1_ exponent was lower in those with long LOS. In contrast, the mean, SD, CV, and ARV of preoperative SBP did not differ between the two subgroups (short *versus* long ICU LOS). The histograms of BPF, mean, SD and ARV of preoperative SBP are shown in Figure 3.

### Associations with cross-sectional age

Preoperative mean SBP, ARV as well as BPF increased linearly with cross-sectional age (Table 2). DFA ⍺_1_ decreased linearly with cross-sectional age (Table 2). The SD and the CV of SBP did not show a statistically significant relationship with age. Overall, BPF was the metric with the strongest association with age (Pearson correlation coefficient: 0.34, *p* < 0.001).

### Associations with the STS and EuroSCORE II risk indices

Preoperative BPF and ARV were positively associated with the STS and the EuroSCORE II risk indices (Table 3). DFA ⍺_1_ was negatively associated with these indices (Table 3). The mean, SD and CV indices of SBP were not associated with the surgical risk scores.

### Associations with surgical ICU LOS

In analyses in which LOS was analyzed as a continuous variable, increased preoperative BPF was significantly associated with longer ICU stay (Table 4). Higher ARV and lower DFA ⍺_1_ values were also associated with longer ICU stay but statistical significance was not reached in the more adjusted models. Adding ARV or DFA ⍺_1_ to any of the models did not change the strength of the associations between BPF and ICU LOS (results not shown). Notably, BPF was the only preoperative dynamical measure that added value to the surgical risk indices in the prediction of long ICU LOS.

In analyses in which ICU LOS was analyzed as a dichotomous variable, increased preoperative BPF was also significantly associated with long ICU LOS in all models (Table 5). Specifically, a one-SD increase in preoperative BPF (9%) was associated with a 26% (13%–40%) (unadjusted analyses) higher likelihood of long ICU LOS (Table 5). The association was slightly attenuated in analyses adjusted for age and sex. To put the strength of these associations in perspective, we note that a one-SD increase in the logarithm of the STS risk index (0.61%) was associated with a 30% (17%–44%) higher likelihood of long ICU LOS. Preoperative mean, SD, CV and ARV as well as DFA ⍺_1_ were not associated with long ICU LOS in adjusted models (Table 5).

Table 6 shows the relative risk of long ICU LOS for all the variables in two different models. Model A included STS, BPF and ARV. Model B included EuroSCORE II, BPF and DFA ⍺_1_. In Model A, a one-SD increase in BPF was associated with a 15% (3%–29%) increase in the likelihood of long ICU LOS. The results for Model B were almost identical, 15% (3%–28%). Neither ARV nor DFA ⍺_1_ was associated with long ICU LOS.

**TABLE 6:** Two specific modified Poisson regression models of long ICU LOS.

MODEL A				
Independent Variables	RR	95% CI		*p* value
STS Morbidity/Mortality (%)	1.22	1.09	1.36	**0.001**
BPF (%)	1.15	1.03	1.29	**0.014**
ARV (mmHg)	1.05	0.96	1.15	0.257

MODEL B				
Independent Variables	RR	95% CI		*p* value
EuroSCORE II (%)	1.24	1.12	1.38	**< 0.001**
BPF (%)	1.15	1.03	1.28	**0.012**
DFA ⍺1	1.01	0.90	1.13	0.889

The values shown are the regression coefficients (ß) and 95% CIs, for standardized variables. Statistically significant *p* values are highlighted in bold. Abbreviations: ARV, average real variability; BPF, blood pressure fragmentation; CI, confidence interval; DFA, detrended fluctuation analysis; EuroSCORE II, European System for Cardiac Operative Risk Evaluation; ICU, intensive care unit; LOS, length of stay; RR, relative risk; and STS, Society of Thoracic Surgeons.

Finally, we re-ran all the analyses including participants previously excluded (see Methods). The results were qualitatively the same. Tables 4, 5 derived from analyses of the entire cohort are provided in the Supplementary Material.

The scatter plots with regression lines and their 95% confidence intervals for BPF *versus* age, STS *versus* BPF, and the postoperative number of hours in the ICU *versus* BPF are also shown in the Supplementary Material.

## Discussion

This study introduces the concept of blood pressure fragmentation, BPF, as the basis of a new dynamical approach for assessing cardiovascular dysregulation. As support of principle, in a population of patients undergoing elective major cardiac surgery we show that increased preoperative BPF was strongly associated with: (i) older age, (ii) increased surgical risk, quantified using the two most widely used scores, the STS surgical risk of morbidity and mortality and the EuroSCORE II, and (iii) longer ICU LOS. Furthermore, we show that preoperative BPF added value to the risk scores in the prediction of ICU LOS.

BPF is perhaps most readily conceptualized as a continuous dynamical property whose extreme manifestation is sustained pulsus alternans, a distinctive pathologic hemodynamic pattern defined by a repetitive beat-to-beat change in the amplitude of the BP waveform (high-low-high-low). Credit for the initial recognition of pulsus alternans is attributed to Ludwig Traube in 1872 (Traube, 1872). This BP pattern has been shown to be associated with chronic heart failure syndromes, particularly in the context of reduced left ventricular ejection fraction (Surawicz and Fisch, 1992; Cha and Falk, 1996). The precise mechanisms of pulsus alternans are not yet fully understood. However, at the subcellular/molecular level, pulsus alternans has been shown to be associated with altered calcium ion cycling within the contractile apparatus of dysfunctional ventricular myocytes (Qu et al., 2013). To the extent that BPF represents a generalization of pulsus alternans, some etiologies should be common to both.

In healthy physiology, gradual beat-to-beat fluctuations in BP (Figure 1) are primarily attributable to oscillatory changes in cardiac output associated with breathing. At rest, BP typically declines during inspiration (<10 mmHg for SBP) and increases during expiration. These fluctuations are mechanically coupled to the respiratory phase and constitute the fastest oscillation (highest frequency) observed under physiologic conditions (One exception is the exaggerated declines in SBP with inspiration, termed pulsus paradoxus, which occurs under pathologic conditions such as severe asthma and pericardial tamponade.) A second contributor to beat-to-beat fluctuations in BP is the baroreceptor reflex, which is mediated by vagal and adrenergic nerve traffic (Kaufmann et al., 2020).

In contrast, under pathologic conditions, oscillations in BP above the physiologic respiratory and baroreceptor reflex frequencies may emerge (Figure 1). We refer to these oscillations as blood pressure fragmentation. From a dynamical perspective, BPF may be understood as a “generalization” of sustained BP alternans. While only a single dynamical pattern, “high-low-high-low,” meets the strict definition of BP alternans, many less organized variations of this pattern (e.g., high-low-high-high-low-high) contribute to BPF.

We hypothesized that higher degrees of BPF would reflect diminished auto-regulatory capacity. Accordingly, we anticipated that increased BPF, indicating reduced hemodynamic adaptability, would be positively associated with cross-sectional age and with standard preoperative risk scores. Theses hypotheses were confirmed as mentioned above. Additionally, since the predicted risk of adverse outcomes and ICU LOS are themselves correlated, we hypothesized that increased BPF would be associated with longer ICU LOS. We found that preoperative BPF predicted ICU LOS almost as well as STS (The risk ratios for BPF and STS were 1.26 [1.13–1.40] and 1.30 [1.17–1.44], respectively.) We then sought to investigate whether preoperative BPF could enhance the predictive accuracy of the surgical risk scores. Improving the performance of these scores is an important but challenging problem given that they derive from models that already include a comprehensive array of optimally-weighted demographic, surgical and clinical variables. We found that preoperative BPF did in fact add value to the risk scores. Our finding supports the contention that this new dynamical metric quantifies information about an individual’s cardiovascular status not subsumed by the risk scores. In general, dynamical analyses of system-level (“integrative”) signals have been shown to be useful in assessing the functional status of the regulatory networks that control such outputs (Goldberger, A. L., 2006; Costa et al., 2018; Cai et al., 2023; Bakkar et al., 2021; Berger et al., 2022). BP is a physiologic variable controlled by regulatory networks involving the cardiac pump, vascular, neurohumoral and multiple other subsystems (Bakkar et al., 2021; Parati et al., 2023). However, dynamical metrics are still not part of the extensive array of clinical variables or of risk scores. Our findings suggest that incorporation of BPF into statistical or artificial intelligence models might enhance their performance.

The concept of BPF and the metric for its quantification reported here are directly adapted from the construct of heart rate fragmentation (HRF) that we introduced previously (Costa et al., 2017a; Costa et al., 2017b, Costa et al., 2018). Fragmentation analysis was developed to overcome limitations of traditional time and frequency domain metrics of heart rate variability analysis, specifically those assessing high-frequency fluctuations in normal-to-normal sinus intervals. Most likely, neuroautonomic dysregulation is a mechanism that underlies both BPF and HRF. Another possible mechanism of BPF is myocardial dysfunction even in the absence of overt heart failure, the condition most commonly associated with sustained BP alternans. Additionally, the degree of BPF may be augmented by the amplifying effects of increased arterial stiffness on BP fluctuations that commonly accompanies aging, hypertension and atherosclerotic disease (Cohn and Finkelstein, 1992; Zieman et al., 2005; Webb et al., 2022).

In secondary analyses, we sought to compare BPF with both linear and nonlinear measures of BPV in terms of the strength of their associations with age, STS and EuroSCORE II and surgical ICU LOS. The SD and the CV of SBP were not associated with any of the selected outcome metrics. Both the ARV and the DFA ⍺_1_ exponent were associated with the surgical risk indices. However, these dynamical measures were not associated with ICU LOS in the most adjusted models.

An important consideration is that SD, the average dispersion of a set of values relative to their mean, does not take into consideration the temporal ordering of the values. In fact, different sequences of the same set of values, for example, “a b c d e,” “e d c b a,” and “d c b a e,” have exactly the same SD. To help quantify information encoded in the specific temporal ordering of beat-to-beat BP measurements (dynamical information), Mena and others (Mena et al., 2005) introduced the “average real variability (ARV),” defined as the average magnitude of the differences between consecutive BP values. However, ARV also has notable limitations. By definition, ARV does not distinguish an increase in BP from a decrease in BP of the same magnitude, i.e., ARV does not capture information encoded in the derivative (first difference) of BP values. As an example, let *x* be the difference between two consecutive BP measurements. The ARV metric does not distinguish a monotonically increasing sequence, “*x* 2*x* 3*x* 4*x* 5*x* 6*x*” from an alternating sequence: “*x* -*x* *x* -*x* *x* -*x*.” This inherent limitation is important because, as we show in this study, dynamical information related to the temporal order of the increases and decreases in SBP appears to be a clinically relevant biomarker of short-term BP control status.

The DFA ⍺_1_ exponent is sensitive to the temporal ordering of a sequence of data points. In this study, lower DFA ⍺_1_ values tended to be associated with longer ICU LOS, analyzed both as a continuous and as a binary variable. However, in contrast to BPF, the associations did not reach statistical significance in models adjusted for STS or EuroSCORE II risk indices. The relatively poorer performance of DFA ⍺_1_ may be due to the violation of a key assumption, namely, the existence of a linear (log-log) relationship between the fluctuation function and number of data points over short time scales.

### Study limitations

By design, this study was limited to the assessment of BPF in predominantly middle-aged to older subjects undergoing major cardiac surgery. We used only invasive (radial artery) recordings obtained immediately prior to surgery. We note that patients were administered midazolam, which may lower BP and possibly modify BPF. However, mean systolic BP was itself not associated with either one of the surgical risk scores (Table 4) or with ICU LOS (Tables 5, 6). Results based on noninvasive recordings obtained concurrently with the assessment of clinical variables used for the computation of the preoperative surgical risk scores would provide stronger evidence of the potential utility of BPF for risk stratification. Finally, we note that we were unable to determine the association of BPF with 30-day mortality due to the limited number of these events (*n* = 9).

### Future studies

Our findings encourage future prospective studies of other populations. For example, investigations of BPF’s utility for risk stratification in outpatient settings and for assessing interventions designed to improve cardiovascular function in overt or sub-clinical heart failure syndromes will be of interest. Although noninvasive estimation of BP from the photoplethysmogram (PPG) has been proven challenging, there is evidence (Besleaga et al., 2018) that PPG can be used to track fast hemodynamic changes and instabilities. Thus, to the extent that BPF is based on relative, not absolute changes in BP waveform amplitude, fragmentation indices may also be used in analyses of PPG signals. Studies will be needed to determine whether BPF (a single-scale method) adds value to other computational metrics, such as multiscale entropy (Rangasamy et al., 2020; Bakkar et al., 2021) and low-frequency power of BPV (Gruenewald et al., 2023). Future studies will also be needed to probe the underlying mechanisms of BPF.

## Conclusion

We introduce a novel metric of beat-to-beat BPV, termed blood pressure fragmentation, BPF, which quantifies BP dysregulation over short time scales. In a cohort study of middle-aged to older adults undergoing elective cardiac surgery, preoperative BPF was strongly associated with older age, increased surgical risk, and longer ICU LOS. This study provides further evidence that clinically relevant information beyond mean and variance is encoded in beat-to-beat BP fluctuations.

## Data Availability

The original contributions presented in the study are included in the article/Supplementary Material, further inquiries can be directed to the corresponding authors.

## Abbreviations

ARV, average real variability; BP, blood pressure; BPF, blood pressure fragmentation; BPV, blood pressure variability; CV, coefficient of variation; DFA, detrended fluctuation analysis; EuroSCORE, European System for Cardiac Operative Risk Evaluation Score; HRF, heart rate fragmentation; LOS, length of stay; SBP, systolic blood pressure; STS, Society of Thoracic Surgeons.
