# Supplementary material for: Blood pressure fragmentation as a new measure of blood pressure variability: association with predictors of cardiac surgery outcomes
Source: Front Physiol. 2024 Feb 9;15:1277592. doi: 10.3389/fphys.2024.1277592 (PMC10884313; doi:10.3389/fphys.2024.1277592)
Supplement: Supplementary file 1 [file DataSheet1.pdf]

SUPPLEMENTARY MATERIAL

Models of the associations between each preoperative SBP dynamical metric and ICU LOS analyzed both as a continuous (Table A) and as binary variable (Table B) are presented below for the entire study cohort, which includes patients with paroxysmal or persistent atrial flutter or atrial fibrillation during final preoperative preparation (n = 44), electronic pacemaker (n = 10) and those (n = 9) who died within 30 days of surgery given that 33% of them (3 out of 9) had a “misleadingly” short (≤ 2 days) ICU LOS.

Table A. Unadjusted and adjusted linear regression analysis of the associations between each preoperative SBP dynamical metric and postoperative number of hours in the ICU.

A. Sub-group with STS (N = 389)															
	**Model 0: Unadjusted**					**Model 1: Age & Sex**					**Model 2: STS**				
**Variable**	**ß**	**95% CI**		**P value**	**R-squared**	**ß**	**95% CI**		**P value**	**R-squared**	**ß**	**95% CI**		**P value**	**R-squared**
BPF (%)	0.22	0.11	0.31	**< 0.001**	0.048	0.17	0.06	0.29	0.003	0.069	0.10	-0.00	0.19	0.059	0.123
Mean SBP (mmHg)	-0.01	-0.13	0.10	0.809	0.000	0.03	-0.15	0.07	0.486	0.045	-0.00	-0.11	0.10	0.965	0.115
SD (mmHg)	-0.02	-0.11	0.06	0.674	0.000	-0.02	-0.11	0.07	0.668	0.044	0.00	-0.08	0.09	0.923	0.115
CV	-0.02	-0.11	0.08	0.742	0.000	-0.01	-0.09	0.08	0.916	0.044	-0.00	-0.09	0.08	0.971	0.115
ARV (mmHg)	0.13	0.02	0.24	**0.025**	0.034	0.11	-0.00	0.22	0.056	0.066	0.07	-0.03	0.17	0.155	0.125
DFA α_1	-0.21	-0.31	-0.10	**< 0.001**	0.047	-0.16	-0.27	-0.04	**0.007**	0.069	-0.06	-0.17	0.04	0.276	0.118
STS (%)	0.33	0.23	0.42	**< 0.001**	0.115										
B. Group with EuroSCORE II (N = 439)															
	**Model 0: Unadjusted**					**Model 1: Age & Sex**					**Model 2: EuroSCORE II**				
**Variable**	**ß**	**95% CI**		**P value**	**R-squared**	**ß**	**95% CI**		**P value**	**R-squared**	**ß**	**95% CI**		**P value**	**R-squared**
BPF (%)	0.24	0.13	0.33	**< 0.001**	0.049	0.19	0.08	0.29	0.001	0.070	0.14	0.04	0.24	0.006	0.107
Mean SBP (mmHg)	0.01	-0.10	0.12	0.879	0.000	-0.01	-0.12	0.09	0.833	0.042	0.02	-0.08	0.12	0.685	0.092
SD (mmHg)	-0.02	-0.11	0.07	0.637	0.000	-0.02	-0.11	0.06	0.603	0.042	-0.01	-0.09	0.08	0.871	0.092
CV	-0.03	-0.11	0.06	0.581	0.001	-0.01	-0.10	0.07	0.661	0.042	-0.02	-0.11	0.07	0.653	0.092
ARV (mmHg)	0.14	0.03	0.26	**0.015**	0.036	0.12	0.01	0.24	**0.035**	0.068	0.10	-0.01	0.20	0.062	0.109
DFA α_1	-0.22	0.33	-0.11	**< 0.001**	0.048	-0.17	-0.29	-0.06	**0.003**	0.070	-0.10	-0.22	0.01	0.076	0.100
EuroSCORE II (%)	0.31	0.21	0.41	**< 0.001**	0.092										

The values shown are the regression coefficients (ß) and 95% CIs for standardized variables. Separate models were fitted for each of the different BPV indicators. The adjustments were age and sex in Model 1, and STS or EuroSCORE II in Model 2. Statistically significant P values are highlighted in bold. Abbreviations: ARV, average real variability; BPF, blood pressure fragmentation; CI, confidence interval; CV, coefficient of variation; DFA, detrended fluctuation analysis; EuroSCORE, European System for Cardiac Operative Risk Evaluation; ICU, intensive care unit; LOS, length of stay; SBP, systolic blood pressure; SD, standard deviation; and STS, Society of Thoracic Surgeons.

Table B. Unadjusted and adjusted modified Poisson regression analyses of the associations between each preoperative SBP dynamical metric and long ICU LOS.

A. Sub-group with STS (N = 389)												
	**Model 0: Unadjusted**				**Model 1: Age & Sex**				**Model 2: STS**			
**Variable**	**RR**	**95% CI**		**P value**	**RR**	**95% CI**		**P value**	**RR**	**95% CI**		**P value**
BPF (%)	1.24	1.12	1.37	**< 0.001**	1.17	1.05	1.31	**0.005**	1.15	1.04	1.26	**0.008**
Mean SBP (mmHg)	0.98	0.89	1.09	0.723	0.95	0.86	1.05	0.351	0.99	0.90	1.08	0.818
SD (mmHg)	0.96	0.86	1.06	0.398	0.96	0.86	1.06	0.385	0.97	0.88	1.08	0.578
CV	0.96	0.86	1.06	0.425	0.97	0.87	4.00	0.561	0.97	0.87	1.07	0.530
ARV (mmHg)	1.08	1.01	1.15	**0.019**	1.06	0.99	1.13	0.082	1.05	0.99	1.11	0.123
DFA α_1	0.85	0.78	0.94	**0.001**	0.90	0.82	1.00	**0.040**	0.93	0.84	1.02	0.158
STS (%)	1.25	1.14	1.36	**< 0.001**								
B. Group with EuroSCORE II (N = 439)												
	**Model 0: Unadjusted**				**Model 1: Age & Sex**				**Model 2: EuroSCORE II**			
**Variable**	**RR**	**95% CI**		**P value**	**RR**	**95% CI**		**P value**	**RR**	**95% CI**		**P value**
BPF (%)	1.23	1.12	1.35	**< 0.001**	1.18	1.06	1.30	**0.002**	1.12	1.02	1.24	**0.022**
Mean SBP (mmHg)	1.00	0.91	1.10	0.956	0.98	0.90	1.08	0.737	1.02	0.93	1.11	0.665
SD (mmHg)	0.97	0.87	1.06	0.492	0.96	0.88	1.06	0.454	0.98	0.89	1.08	0.701
CV	0.96	0.87	1.06	0.447	0.97	0.88	1.06	0.489	0.97	0.88	1.06	0.494
ARV (mmHg)	1.08	1.01	1.15	**0.010**	1.06	1.00	1.13	**0.038**	1.04	0.99	1.10	0.113
DFA α_1	0.84	0.77	0.91	**< 0.001**	0.88	0.80	0.96	**0.003**	0.94	0.85	1.03	0.191
EuroSCORE II (%)	1.32	1.22	1.42	**< 0.001**								

The values shown are the relative risk (RR) and 95% CIs for standardized variables. Separate models were fitted for each of the different BPV indicators. The adjustments were age and sex in Model 1, and STS or EuroSCORE II in Model 2. Statistically significant P values are highlighted in bold. Abbreviations: ARV, average real variability; BPF, blood pressure fragmentation; CI, confidence interval; CV, coefficient of variation; DFA, detrended fluctuation analysis; EuroSCORE, European System for Cardiac Operative Risk Evaluation; ICU, intensive care unit; LOS, length of stay; SBP, systolic blood pressure; SD, standard deviation; and STS, Society of Thoracic Surgeons.

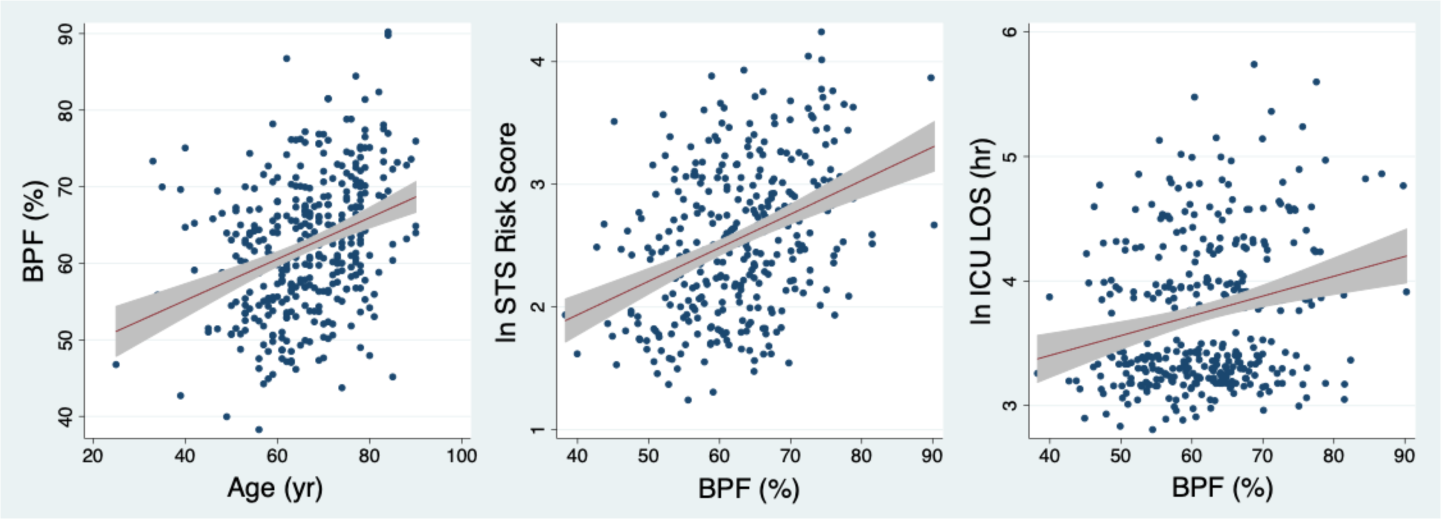

Figure A: Scatter plots with regression lines (red) and their 95% confidence intervals (grey). Preoperative blood pressure fragmentation (BPF) versus age (left); the natural logarithm of the Society of Thoracic Surgeons (STS) risk score versus BPF (center) and the natural logarithm of the postoperative number of hours in the ICU versus BPF (right).
